# SH3BGRL confers innate drug resistance in breast cancer by stabilizing HER2 activation on cell membrane

**DOI:** 10.1186/s13046-020-01577-z

**Published:** 2020-05-07

**Authors:** Hui Li, Mingming Zhang, Yanli Wei, Farhan Haider, Yitong Lin, Wen Guan, Yanbin Liu, Shaoyang Zhang, Ronghua Yuan, Xia Yang, Shulan Yang, Haihe Wang

**Affiliations:** 1 Centre for Translational Medicine, the First Affiliated Hospital, Sun Yat-sen University, 58 Second Zhongshan Road, Guangzhou, 510080 China; 2 Department of Biochemistry, Zhongshan School of Medicine, Sun Yat-sen University, 74 Second Zhongshan Road, Guangzhou, 510080 China; 3 The Second Hospital of Jilin University, Changchun, 130041 China; 4 Institute of Immunology and Molecular Medicine, Jining Medical University, Jining, 272067 Shandong China; 5 Department of General Surgery, The Second Affiliated Hospital of Nantong University, Nantong University, Nantong, 226001 China

**Keywords:** SH3BGRL, HER2, Breast cancer, Drug resistance

## Abstract

**Background:**

HER2-positive breast cancer is usually associated to the more aggressive progression and the worse prognosis, but the mechanism underlying the innate resistance to HER2-targeted therapy remains elusive. The scaffold protein SH3-domain-binding glutamic acid-rich protein-like protein (SH3BGRL) is indicated as a tumor suppressor in some cancers, but it is highly expressed in breast cancers. Here we characterized the tumorigenic function of SH3BGRL in HER2-expressing breast cancer cells and the subsequent effect in HER2-targeted therapies.

**Methods:**

The interaction of SH3BGRL to HER2 were characterized with various truncated SH3BGRL mutants by immunoprecipitation and molecule docking simulation. The physiological roles of SH3BGRL interacting with HER2 in tumor progression and therapy implication were characterized by gain and loss of function approaches in vitro and in vivo. Immunohistochemistry was used for detections of SH3BGRL and p-HER2 (Y1196) expressions in xenografted tumors and human breast cancer tissues. Clinical relevance of SH3BGRL expression with HER2 was validated with both breast patient sample and the public data analyses.

**Results:**

Our results demonstrated that SH3BGRL directly binds with HER2 on cell membrane via its motifs α1, α2 helixes and β3 sheet, which postpones HER2 internalization upon EGF stimulation. Consequently, the association between SH3BGRL and HER2 contributed to the prolonged HER2 phosphorylation at specific tyrosine sites, especially at Y1196, and their downstream signaling activation. The relevance between SH3BGRL expression and p-HER2 (Y1196) phosphorylation was validated in both xenografted tumors and the breast cancer patient tissues. Mechanistically, SH3BGRL promoted breast tumor cell proliferation and survival, while reduced the cell sensitivity to anti-tumor drugs, especially to the HER2-targeted drugs. In contrast, Silencing SH3BGRL or inhibiting its downstream signals efficiently induced apoptosis of breast tumor cells with HER2 and SH3BGRL doubly positive expression. Database analysis also highlighted that SH3BGRL is a poor prognostic marker, especially for HER2-positive breast cancers.

**Conclusions:**

Our results disclose SH3BGRL as a novel posttranslational modulator of HER2 hyperactivation, which can lead to the intrinsic resistance to HER2-targeted therapy. SH3BGRL would be a pivotal therapy target and a diagnostic marker to HER2-positve patients. Thus, targeting SH3BGRL or the downstream signaling could relieve the innate resistance to some HER2-tageted therapies for both HER2 and SH3BGRL-postive breast cancers.

## Background

Breast cancer is the most prevalent malignancy for women worldwide, with 1.67 million new cases were diagnosed in only 2012 [1]. Breast cancer is characterized as a heterogeneous type of disease with various histopathological features and genetic variability, leading to complex prognostic outcomes. This diversity points the importance of understanding the biology of breast cancer and provides more precise and efficient therapy strategy. Now, breast cancers are classified into four major types as luminal A (ER^+^and/or PR^+^, HER2^−^, Ki-67^+^ < 20%), luminal B (ER^+^ and/or PR^+^, HER2^−^, Ki-67^+^ ≥ 20%), luminal B-HER2^+^ (ER^+^ and/or PR^+^, HER2^+^), HER2^+^ (ER^−^PR^−^HER2^+^), and triple-negative (TN; ER^−^PR^−^HER2^−^), based on the molecular profile [2].

HER2, also known as human epidermal growth factor receptor 2, is encoded by the oncogene *Erbb2* [3]. It belongs to the epidermal growth factor receptor (EGFR) family, which contains four members: EGFR (HER1, ErbB1), HER2 (ErbB2, HER2/neu), HER3 (ErbB3), and HER4 (ErbB4). HER2 usually acts as an orphan receptor to be a heterodimer partner to other EGFR members upon growth factor binding, which triggers receptor tyrosine phosphorylation and the downstream kinases activation for intracellular signaling transduction [4]. This signaling renders multiple critical cellular functions, including cell survival, proliferation, polarity change and migration, while the aberrant HER2 upregulation often occurs in about 20–30% of breast cancers as well as ovarian cancers with poor prognosis [5–9].

HER2 upregulation is associated with aggressiveness and worse prognosis of breast cancer. Although the HER2 protein-targeted therapy with the specific antibody Herceptin (trastuzumab) has led to efficient therapy improvement in HER2-possitive patients along with the specific HER2 signal inhibition as well as the antibody-dependent cellular cytotoxicity [10]. But the observed portion of intrinsic resistance or the acquired drug tolerance were easily developed for the later relapse. Thus, it is necessary to elucidate the underlying mechanisms of HER2 overexpression and its hyperactivation in breast cancers, in order to find an effective alternative or combined therapy.

SH3BGRL is a member of SH3BGR family which comprises of SH3BGR, SH3BGRL2, and SH3BGRL3 [11]. SH3BGRL broadly expresses in many human tissues and organs, including bone marrow, heart, lung, liver and kidney [12]. Our recent study thoroughly characterized the general expression patterns of SH3BGR family members during zebrafish embryo development [13]. SH3BGRL encodes a protein of 114 amino acids with a conserved proline-rich PLPPQIF region, which includes both Homer EVH1-binding and SH3-binding motifs [14]. As a scaffold protein, SH3BGRL should play important roles in the protein-protein interaction involved in signal transduction, membrane trafficking, cytoskeletal rearrangements and other key cellular processes [15].

Our previous results unmasked a novel role of mouse SH3BGRL (mSH3BGRL) in driving colorectal cancer metastasis through c-Src activation, but the inverse role of human SH3BGRL as a tumor suppressor [16]. The later study further verified the suppression role of human SH3BGRL in leukemogenesis [17]. Clinically, SH3BGRL is highly upregulated in breast tumors and squamous oral carcinoma, implying its possible tumor-promoting role in these contexts [15, 18, 19]. However, the specific mechanism of SH3BGRL in breast cancer is yet to know. A previous report indicated that SH3BGRL may bind to HER2 [20], but the downstream events regarding the breast cancer occurrence was not addressed. In this study, we intend to thoroughly investigate the interaction between SH3BGRL and HER2 and unveil the exact novel role of SH3BGRL in HER2-positve breast tumors.

## Methods

### Cell lines, reagents and antibodies

HEK293T, MCF-7 and MDA-MB-453 cells were purchased from the Cell Bank of Chinese Academy of Sciences (Shanghai, China). HEK293T and MCF-7 cells were maintained in DMEM HIGH GLUCOSE (Hyclone, Los Angeles, USA) supplemented with 10% fetal bovine serum (Hyclone, Los Angeles, USA) and 1% Penicillin-Streptomycin solution (Hyclone, Los Angeles, USA). MDA-MB-453 cells were maintained in RPMI 1640 (Hyclone, Los Angeles, USA) supplemented with 10% fetal bovine serum and 1% Penicillin-Streptomycin solution. All cells were incubated at 37 °C in a humid incubator containing 5% CO_2_. The medium was changed at alternate days, and cells were split at ration 1:3 when reached 90% confluence. SH3BGRL specific monoclonal antibody was purchased from Santa Cruz. Antibodies, including HER2, p-HER2 (Y1196), p-HER2 (Y877), p-HER2 (Y1221/1222), Akt, p-Akt, ERK, p-ERK, Ki-67, GAPDH, γ-Tubulin and Na+/K + -ATPase were purchased from Cell Signaling Technology (Danvers, USA).

### Clinical samples

Twenty three pairs of breast cancer tissues and the corresponding adjacent counterparts were collected with the informed consents, according to Sun Yat-Sen University health regulation, and the study was further approved by the Research Ethics Committee. Tissue microarrays including 76 HER2-positive breast cancer tissues were purchased from Alenabio Company (*Xi*’*an*, China) under the ethics regulation.

### Plasmid constructs and transfection

The EGFP-SH3BGRL, sh-SH3BGRL and HER2-YFP plasmids were constructed as previously described [16]. To construct HA-tagged SH3BGRL mutants, the mutagenesis PCR was performed with the listed pairs of primers (Supplemental Table S1). Cell transfection was performed with Lipofectamine 2000 (Invitrogen, Waltham, USA) in accordance with the manufacturer’s instructions. SH3BGRL overexpression in MCF-7 cells was achieved with EGFP-SH3BGRL plasmids, and the empty vector was used as control. SH3BGRL knockdown in MDA-MB-453 cells was accomplished with two SureSlencing TM shRNA plasmids mixture for human SH3BGRL (TG309466; Origene, Beijing, China) with GFP label, and the scramble shRNA as control [16]. After transfection, cells were selected with 400 μg/ml G418 (Sigma, St. Louis, USA) (for SH3BGRL overexpression) or 1 μg/ml Puromycin (Sigma, St. Louis, USA) (for SH3BGRL knock-down) for 3–4 weeks, and the single stable cell clones were picked under fluorescence Microscope (Nikon, Tokyo, Japan) to make the stable cell pools.

### Western blotting

After the indicated treatments, cells were lysed in RIPA buffer (50 mM Tris (pH 7.4), 150 mM NaCl, 1% Triton X-100, 1% sodium deoxycholate, 0.1% SDS). Equal amount of protein was loaded and simultaneously subjected to electrophoresis in SDS-polyacrylamide gel and transferred to 0.22 μm pore-sized PVDF membranes (Roche, Basel, Switzerland). Membranes were briefly blocked with 5% skim milk and incubated with the primary antibodies overnight at 4 °C, followed by incubation with the species-matched secondary antibody conjugated with HRP (Cell Signaling Technology, Danvers, USA) for 1 h at room temperature prior to chemiluminescence detection.

### Cell proliferation, cell cycle and anti-cancer drug-induced apoptosis assays

Cells were seeded in 96-well plates at a density of 1000 cells per well. The growth rate of cells was evaluated by using the CCK-8 cell proliferation kit (Dojindo Laboratories, Kumamoto, Japan), according to the manufacturers’ instructions. Cell-cycle analysis was carried out by flow cytometry (Beckman CytoFLEX, California, USA) after propidium iodide staining.

For analysis the role of SH3BGRL in anticancer drug-induced apoptosis, parental MCF-7 and SH3BGRL-overexpressing MCF-7 cells were treated with 10 μM Cisplatin (TOCRIS, Abingdon, OX, UK) for 12 h or with 150 ng Herceptin (Roche, Basel, Switzerland) for 96 h, respectively. Likely, MDA-MB-453 and MDA-MB-453 SH3BGRL knockdown cells were treated with 10 μM Cisplatin for 12 h, with 150 ng Herceptin for 96 h, PI3K/AKT inhibitor LY294002 (CST, Danvers, MA,USA) for 24 h, or Herceptin and ATK inhibitor combination for 48 h, respectively. Cell apoptosis was analyzed using the Annexin V/7-AAD Apoptosis Detection kit (Keygen Biotech, Nanjing, China), and the percentage of apoptotic cells was analyzed by flow cytometer (Beckman CytoFLEX, California, USA). The detailed procedures of all above assays were described as previously [16].

### Cell immunofluorescence staining

Cells were grown at low density on coverslips overnight, washed with PBS three times and fixed with 4% paraformaldehyde for 15 min. After permeabilization with 0.5% Triton X-100 for 20 min, cells were blocked with 5% BSA for 30 min at room temperature. Then the cells were incubated with the specific primary antibody overnight at 4 °C, followed by incubation with secondary antibody: goat anti-mouse Alexa Fluor 488 and anti-rabbit Alexa Fluor 594 (Invitrogen, Waltham, USA) for 1 h at room temperature in the dark. Finally, the samples were mounted with Anti-fade reagent with DAPI (Invitrogen, Waltham, USA). Imaging was processed with confocal fluorescence microscope (Nikon, Tokyo, Japan).

### Immunoprecipitation assay

For co-immunoprecipitation, HEK293T cells were co-transfected with each SH3BGRL mutant along with YFP-HER2 plasmids for 48 h. The transfected cells were harvested and lysed with lysis buffer (20 mM Tris (pH 7.4), 150 mM NaCl, 1% NP-40, 20 mM EDTA). For endogenous interaction of SH3BGRL with HER2, MDA-MB-453 cell lysates were directly used for co-immunoprecipitation. All mentioned cell lysates were incubated with SH3BGRL, HER2 specific antibody or normal mouse IgG overnight at 4 °C respectively, followed by mixing with protein A or protein G Magnetic beads (ThermoFisher, Waltham, USA) for another 3 h on a rotator. All beads were washed with lysis buffer for 6 × 5 min, and the precipitated protein complexes were eluted with loading buffer for western blotting analyses with proper antibodies.

### Cell membrane and cytosol protein extraction

After washing with 1 ml of ice-cold PBS, cells were harvested and lysed with the membrane protein extraction reagent A (Plasma Membrane Protein Extraction Kit, Beyotime, China). After homogenization, the mixture was centrifuged at 700 g for 10 min at 4 °C and the supernatant was further centrifuged at 10,000 g for 30 min at 4 °C. The sediment contains the total membrane protein while the supernatant contains cytosol protein. Further, the pellet was mixed with membrane protein extraction reagent B for 20 min and centrifuged at 10,000 g for 30 min at 4 °C to collect the supernatant.

### Molecular docking analysis of SH3BGRL with HER2

Protein tertiary structures of SH3BGRL(PDB:1U6T) and ERBB2 (PDB:1MFG) were downloaded from the protein structure database, and ZDOCK 3.0.2 was used to perform online molecular docking (http://zdock.umassmed.edu/). Many possible docking modes of SH3BGRL and HER2 were obtained. Based on the ZDOCKScore ranking, the docking mode with the highest score was chosen as the interaction simulation between SH3BGRL and HER2, while the energy stability ranking was also considered. The Pymol software was used to analyze the possible binding sites of amino acids in the two proteins.

### Immunodeficient xenograft mouse tumor model

BALB/c-nude mice (female, 4–5 weeks of age, 18-20 g in body weight) were purchased from Beijing Vital River Laboratory Animal Technology Co., Ltd. (China). All experimental procedures were approved by the Institutional Animal Care and Use Committee of Sun Yat-sen University. Briefly, cells were trypisinized, washed and resuspended in phosphate buffered saline at a density of 10^7^ cells/ml. Seven mice were inoculated subcutaneously with 5 × 10^6^ cells of MD-MBA-453 Vector cells into the right dorsal flanks, and MD-MBA-453 SH3BGRL knockdown cells into the left dorsal flanks, respectively. Similarly, another three mice were inoculated subcutaneously with 5 × 10^6^ MCF-7 parental (Vector) and MCF-7 SH3BGRL cells, respectively. For the growth of the MCF-7 derived tumor, 50 μg estrodiol cypionate (MedChemexpress, NJ, USA) was injected in each mouse once 7 days to sustain the stable estrogen level, till the end of experiment. Mice were sacrificed 30 days after tumor implantation, and the tumors were removed and weighed. For drug treatment, 5 × 10^6^ cells of MD-MBA-453 parental cells (Vector) into the right dorsal flanks, and MD-MBA-453 SH3BGRL knockdown (KD) cells into the left dorsal flanks, respectively. Lapatinib, Herceptin or LY294002 were dosed as 40 mg/kg, 20 mg/kg or 50 mg/kg via i.p. injection twice a week. PBS was used as a negative control. After injection for one week, volumes of the formed tumors were measured once a week. Mice were sacrificed in the fourth week after tumor cell implantation, and the tumors were removed and weighed. GraphPad Prism 5 and paired t test were used for statistical analysis.

### Immunohistochemical assay

All xenografted and patient tissues were fixed with 4% paraformaldehyde overnight at 4 °C, and then embedded in paraffin. The samples were subsequently sectioned into thin slices and mounted on slides, followed by deparaffinization in xylene and rehydration through a series of ethanol-water solutions. Antigen retrieval was carried out by immersing the sections in citrate acid buffer with heating with Microwave oven. Slides were then blocked with 3% hydrogen peroxide to block nonspecific activity. After rinsing, slides were blocked with 5% BSA, and then incubated with SH3BGRL antibody or p-HER2 (Y1196) antibody overnight at 4 °C. Immunohistochemical staining kit (BOSTER Biological Technology, Wuhan, China) was used for color development. Images were captured and confirmed by professional pathologist under microscope (Nikon, Tokyo, Japan). The color intensity of slides was divided into four grades (points) to score SH3BGRL and p-HER2 (Y1196) expression level for statistical analysis.

### Statistical analysis

The SPSS 20.0 were performed for the statistical analysis and the data are presented as mean ± SEM. Comparisons between groups were analyzed using Student’s t-test, chi-square test and Kaplan-Meier for survival analysis (data acquired from THE HUMAN PROTEINS ATLAS). The correlationship between SH3BGRL and p-HER2 was assessed using Spearman correlation analysis. Differences were considered to be statistically significant with *p* < 0.05.

## Results

### SH3BGRL binds to HER2 via its α2 helix and β3 sheet

**Fig. 1:**
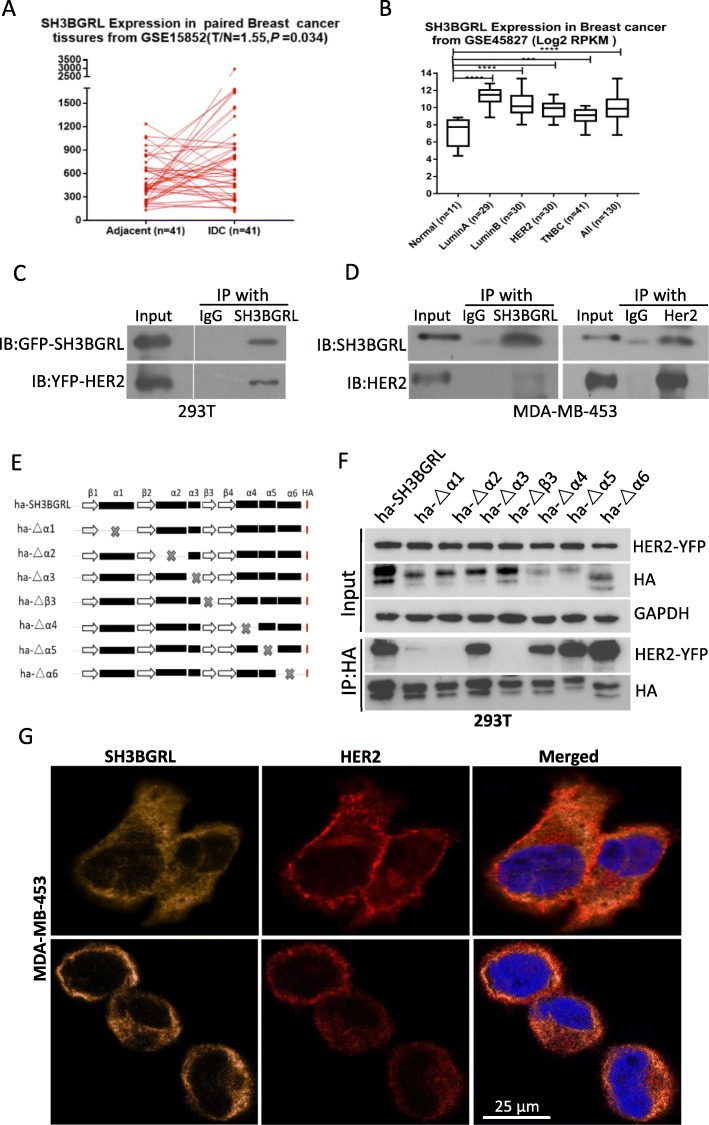
SH3BGRL is related and binds to HER2 via α2 helix and β3 sheet. **a** SH3BGRL expression in invasive ductal carcinoma (IDC), compared to their adjacent normal tissues. Results were analyzed based on the gene microarray dataset, GSE15852. **b** SH3BGRL expression in various types of breast cancers, compared to the normal tissues. Results were analyzed based on the microarray dataset of breast cancer, GSE45827. **c** Interaction between exogenous SH3BGRL and HER2. GFP-SH3BGRL or control vector and YFP-HER2 plasmids were co-transfected into HEK 293 T cells, and then immunoprecipitation (IP) and immunoblotting (IB) were performed with anti-HER2 antibodies. **d** Interaction of endogenous SH3BGRL with HER2 determined by Co-IP analyses in MDA-MB-453 cells. **e** Schematic SH3BGRL truncation mutants based on its structure domains. **f** Co-IP of the HA-SH3BGRL truncation mutants and YFP-HER2. Cells were co-transfected in HEK 293 T cells, and SH3BGRL and HER2 were respectively immunoprecipitated with anti-HA or anti-HER2 antibodies. **g** Confocal analysis of the co-localization of HER2 and endogenous SH3BGRL in MDA-MB-453 cells. Cells were stained with SH3BGRL (yellow), HER2 antibody (red) and merged images are shown on the right

Previous results indicate that human SH3BGRL functions as a tumor suppressor [16, 17], and only the mutated ones promote tumor metastasis and progression. However, accumulating evidence shows that SH3BGRL is highly upregulated in breast tumors [15, 18, 19], implying its possibly oncogenic role in breast cancer progression. However, the exact function of SH3BGRL in breast cancer remains elusive. To investigate whether SH3BGRL is related to breast cancer progression, we analyzed the gene dataset, GSE15852 on breast cancer and observed that the SH3BGRL expression in Invasive Ductal Carcinoma (IDC) is obviously higher than in adjacent normal tissue (*P* = 0.034) (Fig. 1a). Similar analysis of another gene expression datasets, GSE45827 on breast cancer also manifested that SH3BGRL is more highly expressed in various types of breast cancers than that in normal tissues, suggestive of an oncogenic role of SH3BGRL in breast cancers (Fig. 1b). To examine if there is a direct interaction between SH3BGRL and HER2, as HER2 was predicted to interact with SH3BGRL, we firstly co-transfected HEK293T cells with GFP-SH3BGRL and YFP-HER2 plasmids. Immunoprecipitation with SH3BGRL-specific antibody showed that YFP-HER2 was detected in the precipitated complex compared with the control IgG (Fig. 1c), indicating the exogenous interaction of SH3BGRL and HER2. To confirm the intrinsic interaction of SH3BGRL with HER2, we performed the reciprocal immunoprecipitations with anti-SH3BGRL antibody as well as the anti-HER2 antibody in MDA-MB-453 breast cancer cells, respectively. Immunoblotting results clearly demonstrated that the endogenous HER2 was precipitated by the endogenous SH3BGRL; inversely, SH3BGRL was detected in the precipitated complex with HER2-specific antibody, compared to those with the normal control IgG (Fig. 1d). Structurally, SH3BGRL contains two important binding domains, SH3 and EVH1 motifs, which endow SH3BGRL as a scaffold protein in signaling transduction. The tertiary structure of SH3BGRL comprises 6 α helixes and 4 β sheets [21]. In order to characterize which specific binding motif in SH3BGRL bound to HER2, we deleted all 6 α helixes and β3 sheet (core region of SH3B domain) individually to construct 7 SH3BGRL mutant plasmids (Fig. 1e). After co-transfection of HEK293T cells with YFP-HER2 construct, co-immunoprecipitation assays demonstrated that deletion of either α2 helix or β3 sheet in SH3BGRL evidently abolished the interaction between HER2 and SH3BGRL, and deletion of the α1 helix attenuated the interaction to some extent (Fig. 1f), indicating that besides the recognized SH3 domain (β3 sheet), the other two α1, 2 helix are also critical for the interaction of SH3BGRL with HER2.

Moreover, immunofluorescence also manifested the co-localization of HER2 with some endogenous SH3BGRL on cell membrane of MDA-MB-453 cells (Fig. 1g). Our results at least revealed the direct interaction between SH3BGRL and HER2 via α2 helix and β3 sheet.

### SH3BGRL prolongs HER2 duration on cell membrane

**Fig. 2:**
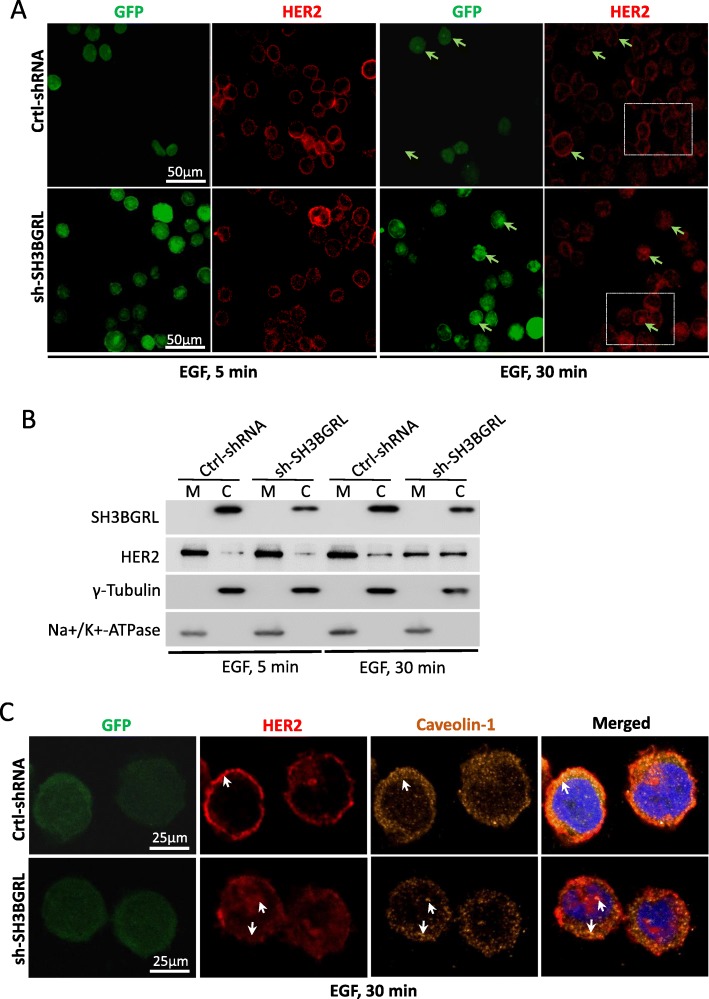
SH3BGRL prolongs the duration of HER2 on cell membrane. **a** Effects of EGF stimulation on intracellular distribution of HER2. Under EGF stimulation (20 ng/mL), HER2 (red) was detected in MDA-MB-453 cells by immunofluorescence assay. Left: EGF stimulated 5 min, Right: EGF stimulated 30 min. Arrows point the HER2 location. Doted boxes show the overall HER2 location states after EGF treatment. **b** Immunoblots of the subcellular fractioned HER2. With EGF stimulation (20 ng/mL), cells were harvested, SH3BGRL and HER2 in cell membrane (M) and cytoplasm (C) fractions were detected. **c** Co-localization of HER2 with the endocytosis-related protein Caveolin-1 in MDA-MB-453 cells by confocal analysis. Cells were stained with Caveolin-1 (orange), HER2 antibody (red), and the merged images are shown on the right. Arrows point the HER2 and Caveolin-1 locations

To address the potential pathological consequence and the impact of the interaction between SH3BGRL and HER2 on HER2-mediated signaling in breast cancer, we established SH3BGRL-overexpressing MCF-7 cells and SH3BGRL-depleted MDA-MB-453 cells (Fig. S1) and performed immunofluorescence staining to investigate the HER2 localization status in MDA-MB-453 cells. With EGF stimulation for 5 min, HER2 was mainly stained on cell membranes in both parental control and MDA-MB-453 SH3BGRL knockdown cells. However, with EGF stimulation for 30 min, HER2 endocytosis was significantly accelerated in SH3BGRL knockdown MDA-MB-453 cells, in contrast, the majority of HER2 still located on cell membrane in parental MDA-MB-453 cells (Fig. 2a). Subcellular fractionization analysis further consolidated the observations of the respective HER2 localizations (Fig. 2b). As HER2 is putatively believed to undergo endocytosis once activated, we used an endosome marker, caveolin-1, to investigate if SH3BGRL depletion affects HER2 location and stability on cell membrane. Staining results revealed that in SH3BGRL-silencing MDA-MB-453 cells, HER2 dispersed into cell cytoplasm along with caveolin-1, whereas in the SH3BGRL highly expressing parental cells, HER2 was still located on the cell membranes and had less endocytosis upon EGF stimulation for 30 min (Fig. 2c). Even without EGF stimulation, HER2 still underwent endocytosis in the SH3BGRL-silencing cells (Supplemental Figure S2). Results here together indicated that the association of SH3BGRL with HER2 sustains the residence of HER2 on cell membrane, even as dimers after growth factor stimulation. Therefore, binding of SH3BGRL to HER2 could block HER2 internalization and prolong HER2 activation on cell membrane.

### SH3BGRL binds to HER2 to enhance tyrosine site-specific phosphorylation of HER2

**Fig. 3:**
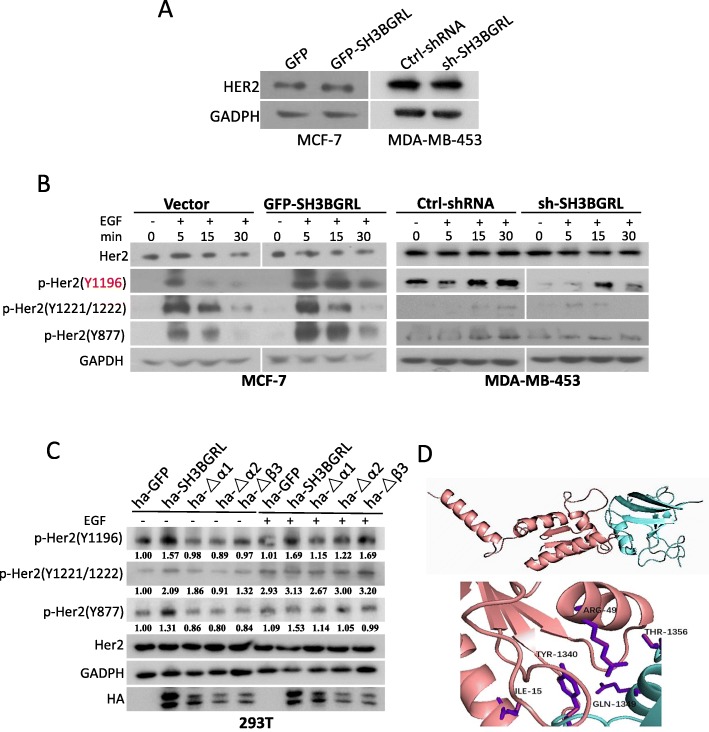
SH3BGRL enhances tyrosine site-specific HER2 phosphorylation. **a** Immunoblots of HER2 in SH3BGRL-overexpressing and knocking down cell lines. **b** Immunoblots of the indicated HER2 phosphorylation by SH3BGRL. SH3BGRL enhances EGF-induced HER2 tyrosine phosphorylation and prolongs the activation time, especially at pHER2 (Y1196) site. Cells were starved for 24 h, and stimulated with 20 ng/ml EGF for different time points (0, 5, 15 and 30 min). **c** Phosphorylation analysis of HER2 by western blotting assay. Transient co-transfection experiments were performed in HEK293T cells with YFP-HER2 and HA-SH3BGRL truncated mutants △α1, △α2, △β3. GAPDH was used as loading control. **d** Molecule docking prediction of the interaction between SH3BGRL and HER2. The model image was generated by Pymol. The upper panel shows the overview interaction of SH3BGRL (in purple) with C-terminal of HER2 (in blue). The lower panel presents the closest amino acid binding sites

As an orphan receptor, HER2 undergoes dimerization with other EGFR family members to initiate varied downstream intracellular signals after growth factor stimulation [4]. To understand the binding consequence of SH3BGRL to HER2, we first determined the dynamic phosphorylation state of HER2. Results showed that with SH3BGRL expression level change, HER2 expression did not present any evident alteration in both MCF-7 and MDA-MB-453 cells, although MCF-7 cell is considered as a HER2-negative cell, but the basal HER2 expression was detected (Fig. 3a). We then treated all those cells starved for 24 h with EGF in a time course manner of 0, 5, 15 and 30 min to characterize the dynamic phosphorylation situation of HER2 along with SH3BGRL expression change. Immunoblotting results showed that SH3BGRL overexpression manifestly prolonged HER2 phosphorylations at Y1196 and Y877 sites in MCF-7 cells (Fig. 3b, left panel). In both the endogenous HER2 and SH3BGRL positive MDA-MB-453 cells, prior to EGF stimulation, HER2 already showed the Y1196 phosphorylation, which further increased by EGF stimulation for long time; in contrast, when knocking down the endogenous SH3BGRL with specific shRNAs, the endogenous HER2 was not phosphorylated at this site, but could be re-phosphorylated with EGF stimulation for 15 min and then be inactivated even with EGF stimulation for 30 min (Fig. 3b, right panel). Meanwhile, HER2 phosphorylation at Y1221/1222 sites was not impaired with SH3BGRL expression level change in both MDA-MB-453 and MCF-7 cells (Fig. 3b).

We further verified the possible influence of SH3BGRL mutation on HER2 phosphorylation through transient co-expression of HA-SH3BGRL and GFP-HER2 in HEK 293 T cells. Consistent with the binding capacity of SH3BGRL mutants with HER2 above, SH3BGRL overexpression could promote HER2 activation, regardless of EGF stimulation. But destroying the SH3 domain in SH3BGRL by deleting the helixes α1, α2, or β3 clearly abolished the wild-type SH3BGRL function on HER2 activation (Fig. 3c). Further molecule docking of SH3BGRL with C-terminal of HER2 revealed that helixes α1, α2, or β3 in SH3BGRL were critical for their interaction, and this interaction may sustain a particular HER2 conformation (Fig. 3d), leading to the tyrosine site-specific HER2 phosphorylation, for instance, at tyrosine 1187.

### SH3GRL enhances HER2 downstream signaling and tumorigenesis

**Fig. 4:**
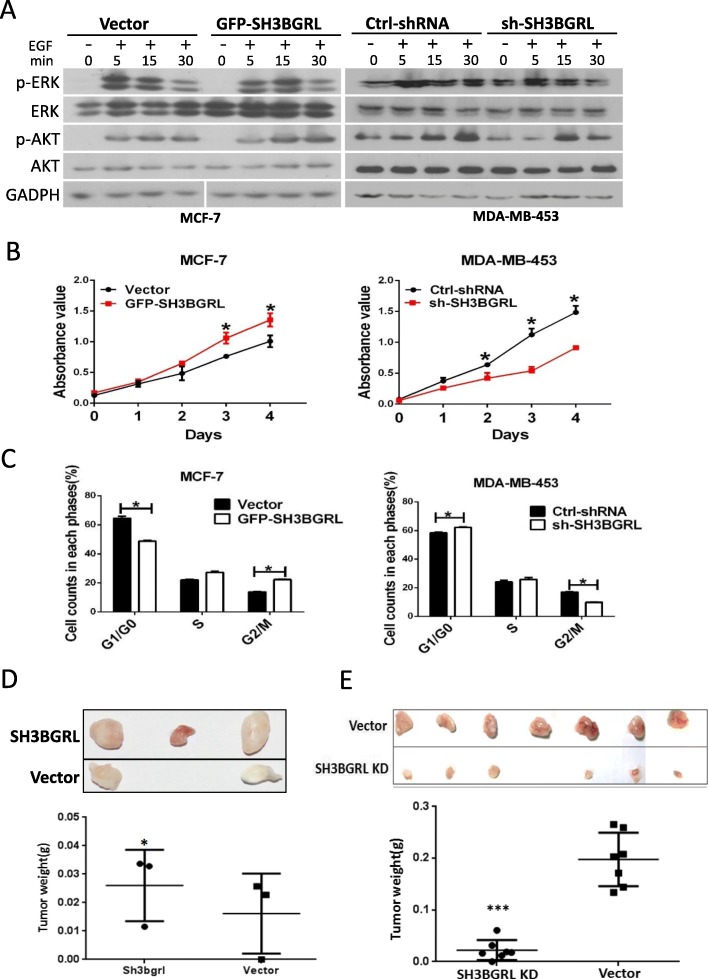
Effect of SH3BGRL on breast cancer cell proliferation, cell cycle and apoptosis. **a** Dynamic analysis of SH3BGRL in downstream AKT and MAPK signaling pathways. The indicated cells were starved for 24 h, and treated with 20 ng/ml EGF for different time points (0, 5, 15 and 30 min), and the indicated proteins were immunoblotted. **b** Cell proliferation of MCF-7 cells by SH3BGRL ectopic expression and MDA-MB-453 cells by SH3BGRL knockdown were assayed by CCK8 methods. **c** Cell cycle progression of MCF-7 and MDA-MB-453 cells are shown. The right side is a representative flow cytometry histogram with the cell population percentage in each cell cycle phase presented as mean ± SEM. **d** Tumor formation induced by MCF-7 cells (Vector) and SH3BGRL overexpression cells (SH3BGRL), respectively. Cells were injected subcutaneously into flank sides of nude mice armpit. Tumors induced were harvested and weighed. The xenograft tumor weights were analyzed and shown in the lower panel, respectively, *n* = 3. **e** Tumor formation induced by MDA-MB-453 cells (Vector) and SH3BGRL knockdown cells (SH3BGRL KD). Cells were injected subcutaneously and analyzed as above. *P* < 0.0001, *n* = 7

HER2 activation is closely with the downstream signaling activation, typically the MAPK and PI3K/AKT pathways. We then evaluated the activation status of PI3K/AKT and MAPK pathways in the correspondingly SH3BGRL-modulated HER2 activation. As expected, SH3BGRL overexpression prolonged activations of AKT and ERK in MCF-7 cells in comparison with the parental MCF-7 cells; however, SH3BGRL knockdown compromised the activation periods of AKT and ERK in MDA-MB-453 cells (Fig. 4a). Taken together, these results indicated that once binding to HER2, SH3BGRL could initiate HER2 activation, prolong the activation duration and the functioning time of the downstream signaling.

To seek whether SH3BGRL involving in the HER2-stimulated cell growth and survival, cell proliferation, cell cycle progression and apoptosis were examined in both MCF-7 and MDA-MB-453 cells. Results showed that SH3BGRL ectopic expression significantly promoted MCF-7 cell proliferation. On the other side, SH3BGRL knockdown suppressed cell proliferation in MDA-MB-453 cells (Fig. 4b). Likely, SH3BGRL overexpression in MCF-7 cells notably promoted cell cycle progression, manifesting the reduced cell population in G0/G1 phase and the increased in G2/M phase; while SH3BGRL silence in MDA-MB-453 cells conversely arrested cell cycle progression, showing the higher cell population at G0/G1 phase and the less in G2/M phase (Fig. 4c, Supplemental Figure S3). Thus, SH3BGRL was verified to promote breast tumor cell cycle progression. To further investigate the physiological effect of SH3BGRL in tumorigenesis, we subcutaneously inoculated SH3BGRL-overexpressing MCF-7 cells with their parental cells into immune-deficient mice. In vivo results showed that SH3BGRL overexpression could efficiently promote tumorigenesis (Fig. 4d). In contrast, when knocking down the endogenous SH3BGRL in MDA-MB-453 cells, the tumorigenesis was significantly repressed, compared to the control cells (Fig. 4e), verifying the oncogenic role of SH3BGRL in breast tumor progression.

### SH3BGRL renders intrinsic HER2-targeted therapy resistance

**Fig. 5:**
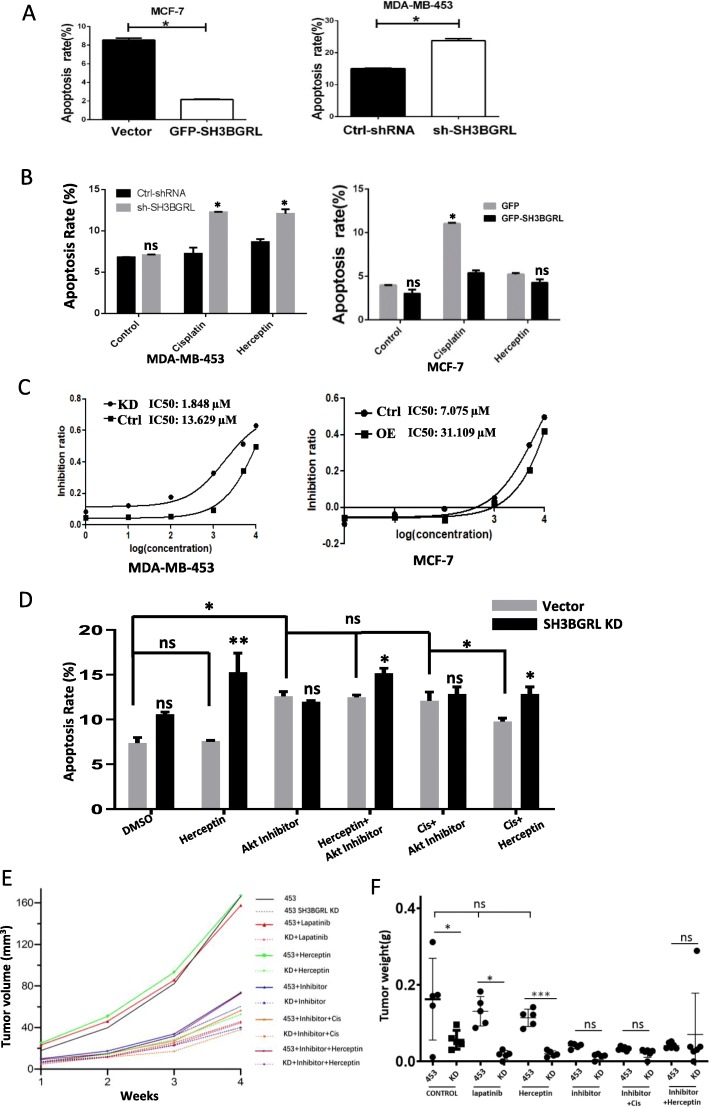
SH3BGRL enhances anti-cancer drug resistance in breast cancer cells. **a** Flow cytometry analysis of the indicated apoptotic MCF-7 and MDA-MB-453 cells with Annexin V/7-AAD staining upon Cisplatin treatment. **b** Flow cytometry analysis of the indicated apoptotic MCF-7 cells with or without SH3BGRL overexpression or apoptotic MDA-MB-453 cells with or without endogenous SH3BGRL knockdown after Herceptin and Cisplatin treatments at the indicated dose and time. **c** IC_50_ analysis of Lapatinib in MCF-7 cells with or without SH3BGRL overexpression, or MDA-MB-453 cells with or without endogenous SH3BGRL knockdown. **d** Apoptotic analyses of the indicated MDA-MB-453 cells with or without endogenous SH3BGRL knockdown by Herceptin (200 μg, 96 h), AKT inhibitor LY294002 (Inhibitor, 40 μM, 24 h), the combination of Herceptin (200 μg) and AKT inhibitor LY294002 (40 μM, 24 h) for 48 h, the combination of Cisplatin (10 μM) and AKT inhibitor LY294002 (40 μM, 24 h) for 12 h, and the combination of Cisplatin (10 μM) and Herceptin (200 μg, 96 h) for 12 h. **e** Tumor growth curve induced by MDA-MB-453 cells (Vector) and SH3BGRL knockdown cells (SH3BGRL KD). Cells were injected subcutaneously, after one week injection, mice were cured with Lapatinib, Herceptin, LY294002, combination of LY294002 and Cisplatin, combination of Herceptin and LY294002 or DMSO via intraperitoneal injection twice a week. **f** Tumor weights with MDA-MB-453 cells (Vector) and SH3BGRL knockdown cells (SH3BGRL KD). Cells were injected subcutaneously and analyzed as above. All experiments were independently conducted for three times and data are shown as mean ± SEM. ^*^p < 0.05; **p < 0.01; ***p < 0.001; ns means no statistical significance

Considering that the HER2-targeted therapy is not universally effective on HER2-possitive breast cancers, we further investigated if SH3BGRL overexpression can interfere with the anti-tumor drug sensitivity in breast cancer cells. We first tentatively determined the impact of SH3BGRL on cisplatin sensitivity. Flow cytometry analysis showed that SH3BGRL overexpression significantly compromised the cisplatin-induced cell apoptosis in MCF-7 cells, while SH3BGRL knockdown sufficiently enhanced cell apoptosis in MDA-MB-453 cells (Fig. 5a; Supplemental Figure S4, Cisplastin), indicating the dual effects of SH3BGRL in breast tumor cell proliferation and anti-cancer drug resistance.

Given the persistent activation of HER2 by SH3BGRL, we sought to figure out whether SH3BGRL can orchestrate the HER2-targeted therapy. We then treated the putative HER2-negative MCF-7 cells with Herceptin. Results manifested that Herceptin did not induce so obvious cell apoptosis in both parental MCF-7 and SH3BGRL-overexpressing MCF-7 cells, compared to that by Cisplatin (Fig. 5b; Supplemental Figure S4, Herceptin). This result hints that even the marginal HER2 is activated with SH3BGRL on cell membrane, the HER2-trageted therapy is still ineffective, implying that Herceptin-based on therapy is mainly effective to the tumors with quite high HER2 expression. To further confirm this observation, we treated MDA-MB-453 cells with both HER2 and SH3BGRL in high level and noted the evident resistance of these cells to Herceptin treatment. In contrast, with SH3BGRL knocked down, those cells became to be the HER2-high plus SH3BGRL-negative/or low MDA-MB-453 cells, and Herceptin in turn promoted these cells to undergo apoptosis as cisplatin (Fig. 5b).

To further check the effect of SH3BGRL to HER2 kinase-based therapy, we treated MDA-MB-453 and MCF-7 cells with a HER2 kinase inhibitor, Lapatinib. Results indicated that endogenous SH3BGRL knockdown in MDA-MB-453 cells sensitized cell sensitivity to Lapatinib, while SH3BGRL overexpression in MCF-7 cells significantly enhanced cell resistance to Lapatinib (Fig. 5c), indicating that SH3BGRL enhances the HER2-positive breast cancer cells resistance to Cisplatin and the HER2-targeted therapies.

As the interaction between SH3BGRL and HER2 activated the downstream cell survival signaling, we sought to know if blocking this signaling transduction pathway, whether can neutralize the effect of SH3BGRL on drug resistance. We thus treated MDA-MB-453 cells with a PI3K inhibitor, LY294002 and found a generally slight increase of cell apoptosis, which is not dependent on SH3BGRL level in MDA-MB-453 cells (Fig. 5d). As PI3K-AKT signaling plays universal roles in cell survival, inhibition this signaling should naturally renders cell death to some extent.

We next approached to the combinational drug treatments of MDA-MB-453 wild-type cells and the SH3BGRL-silencing cells and found that the combination of Herceptin+AKT inhibitor did not presented the synergetic effect on SH3BGRL and HER2 doubly positive MDA-MB-453 cells, compared to AKT inhibitor alone (Fig. 5d-Vector), whereas SH3BGRL KD sensitized those cells to the combined treatment (Fig. 5d-Vector). In the combination of Cisplastin and AKT inhibitor, as both components are not specific to HER2 inhibition, there was no difference between SH3BGRL-expressing and SH3BGRL-delpleting cells to the treatment, but presenting notable cytotoxicity, compared to the DMSO-treated control groups. Moreover, combination of Cisplatin+AKT inhibitor showed more apoptotic effect than Cisplatin+Herceptin on the wild-type MDA-MB-453 cells (Fig. 5d), suggesting the involvement of the SH3BGRL/HER2-mediated AKT activation to drug resistance. Overall, results above confirmed that SH3BGRL endows HER2-positive cells to be resistant to Cisplastin and HER2-specific drugs.

To validate the individual or combination therapy potential of the above assayed drugs, we first inoculated the parental MDA-MB-453 cells and the SH3BGRL knockdown cells into immunodeficient nude mice till the visible tumors formed. Therapy results showed that HER2-trageted Herceptin and Lapatinib did not show any inhibitive role in SH3BGRL and HER2 doubly positive tumors, but AKT inhibitors LY294002 showed the evident inhibition to this doubly positive type tumor progression. Therefore, combinations with AKT inhibitor with Cisplatin or Herceptin exhibited similar inhibitory effects to AKT inhibitor alone. As expected, SH3BGRL knockdown dramatically increased the cell sensitivity to single Lapatinib or Herceptin, leading to the drastic tumor growth inhibition (Fig. 5e, f; Supplemental Figure 5). Furthermore, we examined the tumor cell proliferation and apoptosis states in the various treated tumors with cell proliferation marker Ki67 and the apoptotic TUNEL stainings (Supplemental Figure 6). Staining results demonstrated the matchable trends of tumor cell proliferation and cell death to the corresponding tumor growths and tumor weights upon the treatments. Therefore, targeting SH3BGRL would be a promising strategy to treat the SH3BGRL and HER2 doubly positive breast cancers and release HER2-targeted therapy resistance. Alternatively, targeting SH3BGRL/HER2 downstream signals might be another practical strategy to SH3BGRL high breast cancers, regardless of HER2 expression level.

### Relevance of SH3BGRL with p-HER2 (Y1196) in breast cancer tissue

To verify the HER2 and downstream AKT activation events in the SH3BGRL-positive and SH3BGRL-low xenografted tumors with or without the abovementioned drug treatments, we performed the immunochemistry staining of the sections of xenografted tumors. Staining results confirmed that SH3BGRL knockdown or the effective therapies efficiently repressed HER2 phosphorylation at both Y877 and Y1196 sites, as well as the downstream AKT activation (Supplementary Figure S7), validating the authenticity of SH3BGRL/HER2 tumorigenic mechanism.

**Fig. 6:**
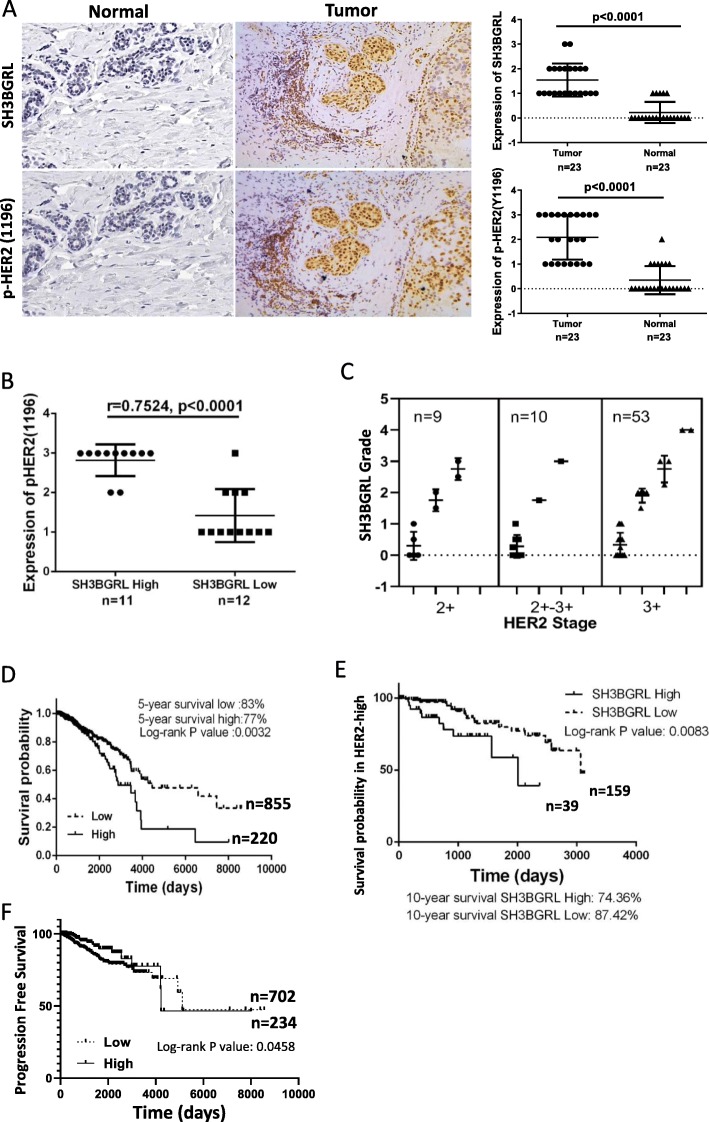
Relevance of SH3BGRL with pHER2 (Y1196) in breast cancer tissues. **a** Representative immunohistochemistry staining of SH3BGRL and p-HER2 (Y1196) expression in serial sections (left panel), and the statistical analyses of their expressions in tumor tissues and their matched adjacent ones from breast cancer patient. **b** Spearman correlation analysis showed that there is a significant positive correlation between the expression of SH3BGRL and pHER2 (Y1196): r = 0.7524, *p* < 0.0001. **c** Statistical analysis of 76 HER2-positive breast cancer cases in tissue microarray after immunohistochemistry detection of SH3BGRL and HER2 expression. **d** Kaplan-Meier survival analyses of SH3BGRL expression to 1075 breast cancer patients from THE HUMAN PROTEIN ATLAS. The log-rank test *p* values are shown. **e** Kaplan-Meier survival analyses of the 10-year survival rate in 198 patients with high HER2 expression from above dataset. The log-rank test p values are shown. **f** Kaplan-Meier survival analyses of 936 patients from above TCGA Pan-Cancer Clinical Data Resource data with SH3BGRL expression and the progression free survival rate. The log-rank test p values are shown

To further validate the relevance of SH3BGRL on breast cancer progression in patients, we collected 23 pairs of fresh clinical breast cancer samples and performed the immunohistochemistry analysis again, in which the protein staining intensity was divided into four grades by double-blind scoring (Supplemental Figure S8A). Quantitative expression analysis showed that SH3BGRL and p-HER2 (Y1196) expression were both significantly higher in tumor tissues than those in the adjacent normal tissues (Fig. 6a). Statistically, there was a clear correlation (r = 0.7524, *p* < 0.0001) between SH3BGRL and p-HER2 in paired tissues (Fig. 6b). We then utilized a breast tissue array with 72 HER2-positive samples to analyze SH3BGRL expression level and showed that SH3BGRL is highly expressed in up to 60% of HER2-positive patients, although with the limited case amount (Supplementary Figure 8B,C). In addition, patients with strongest SH3BGRL expression also overlapped with the highest HER2 level cases (Fig. 6c).

To scale up the case number, we analyzed 1075 breast cancer patients data in THE HUMAN PROTEIN ATLAS database. The relationships between SH3BGRL expression and various clinical features were summarized in Table 1). All data were divided into two groups by SH3BGRL expression level cutoff (232.4 FPKM), and the plotted Kaplan-Meier survival curve demonstrated that high SH3BGRL expression is strictly associated with the poor survival of patients (Fig. 6d, *p* < 0.0032). These patients were further subdivided into HER2-high and -low groups, with a 10-year survival analysis, we found high SH3BGRL expression was still a poor prognostic marker in HER2-high subgroup (Fig. 6e), but no such information to the HER2-low subgroup (Supplemental Figure 8D). Furthermore, data from TCGA Pan-Cancer Clinical Data Resource showed that high SH3BGRL expression was related to poor progression-free interval (PFI) (Fig. 6f). These results together manifested that SH3BGRL plays critical oncogenic function in HER2-high breast cancers, and SH3BGRL expression status is a potent biomarker to subgroup HER2-positive breast cancers and the suitable therapy strategy.

**Table 1:** Association between SH3BGRL expression and the clinical features in 1075 breast cancer patients

		Expression		
Variables	Cases (%)	High	Low	***P*** value
**Gender**				
Female	1063 (98.9%)	218 (20.5%)	845 (79.5%)	0.74
Male	12 (1.1%)	2 (16.7%)	10 (83.3%)	
**Status**				
Alive	923 (85.9%)	177 (19.2%)	746 (80.8%)	0.01
Dead	152 (14.1%)	43 (28.3%)	109 (71.7%)	
**Age (Years)**				
≥ 50	775 (72.1%)	170 (21.9%)	605 (78.1%)	0.143
< 50	286 (26.6%)	47 (16.4)	239 (83.6%)	
unknown	14 (1.3%)	3 (21.4%)	11 (78.6%)	
**Stage**				
I	89 (8.3%)	19 (21.3%)	70 (78.7%)	0.17
Ia	86 (8.0%)	24 (27.9%)	62 (72.1%)	
Ib	5 (0.5%)	0 (0.0%)	5 (100.0%)	
II	6 (0.6%)	0 (0.0%)	6 (100.0%)	
IIa	352 (32.7%)	82 (23.3%)	270 (76.7%)	
IIb	251 (23.3%)	41 (16.3%)	210 (83.7%)	
III	2 (0.2%)	0 (0.0%)	2 (100.0%)	
IIIa	153 (14.2%)	32 (20.9%)	121 (79.1%)	
IIIb	25 (2.3%)	4 (16.0%)	21 (84.0%)	
IIIc	63 (5.9%)	12 (19.0%)	51 (81.0%)	
IV	20 (1.9%)	2 (10.0%)	18 (90.0%)	
X	11 (1.0%)	0 (0.0%)	11 (100%)	
unknown	12 (1.1%)	4 (20.5%)	8 (79.5%)	

## Discussions

Intrinsic resistance of HER2-targeted therapy limits the survival of a portion of breast cancer patients, but the behind mechanism remains elusive. Protein interactomic landscape of HER2-positive breast cancers predicts that SH3BGRL may relate to HER2, but no any information on the physiological function of their interplaying was reported [20]. Here we evidently characterized that SH3BGRL directly binds to HER2 via its motifs α1, α2 helixes and β3 sheet (core proline-rich PLPPQIF of SH3 domain) on cell membrane of breast tumor cells. This association stabilizes HER2 on cell membrane through delaying the endocytosis of HER2-containing dimers of EGFR family as well as the dimerization without ligand stimulation. This phenomenon subsequently contributes to the prolonged HER2 activation and function duration of the downstream signaling in breast tumor cells. Consequently, it results in the enhanced tumor cell proliferation and survival ability for breast tumor progression. Besides, this association between HER2 and SH3BGRL also provokes the intrinsic HER2-targeted therapy resistance.

Interestingly, we disclose the function of SH3BGRL in maintaining HER2 on cell membrane, which expands our knowledge in the delicate HER2 regulation in nature, but the detailed mechanism is yet to be resolved. Based on our results, we hypothesize that binding of SH3BGRL to HER2 may block some crucial interacting motifs of HER2 to clathrin or other factors on cell membrane, such as vacuole. Their interaction then leads to the insufficient endocytic vesicle formation for the consequent dimer internalization, as clathrin is necessary to HER2 endocytosis and intracellular transport [22]. In this regard, our result may propose a novel mechanism underlying over-activated HER2 signaling in SH3BGRL-proficient breast cancer.

HER2 is well known as a dimerization partner with other EGFR family members, leading to specific tyrosine phosphorylation in its intracellular kinase region [4]. Different partnership or growth factor stimulation may contribute to different tyrosine site phosphorylation, but no a confirmed example was reported. Here we demonstrated that binding of SH3BGRL to HER2 specifically enhanced the phosphorylation at Y877 in response to EGF stimulation, and Y1196 even without EGF stimulation in HER-2 high breast cancer cells. In parallel, SH3BGRL imposed no obvious effect on Y1221/1222 phosphorylations which were also important for downstream signaling. Therefore, we predict that SH3BGRL may function as a fine switcher by binding the specific HER2 domain(s) to discriminate HER2 signals from other stimulator through the specific tyrosine phosphorylation, which might provide a new way for blocking the specific tyrosine phosphorylation and the particular downstream signaling. Putatively, we speculate that binding of SH3BGRL to HER2 may cause HER2 conformation change to lead to the unique tyrosine phosphorylation maintenance.

Ras/Raf/MAPK and PI3K/AKT pathways are believed to be the downstream signaling triggered by HER2 phosphorylation [23–27]. Consistently, we showed that SH3BGRL stimulated the AKT and ERK phosphorylation through the prolonged HER2 phosphorylation at Y1196, while silencing SH3BGRL abolished these effects, indicating the important effect of SH3GRL in HER2 Y1196 phosphorylation-dependent signaling in breast tumor cells. Targeting the precise HER2 phosphorylation site, such as p-Y1196, would be an effective and straightforward strategy, while can avoid the side effect from the non-selective inhibition of the necessary function of HER2. Therefore, SH3BGRL would be such a candidate. It is known that the appropriate HER2 phosphorylation at the particular tyrosine site is crucial to the normal cell metabolism and function, including breast, ovary tissues.

Given that SH3BGRL only structurally contains two adaptor domains, SH3 binding and homer EVH1 binding motifs, it should be an instinctive adaptor protein to link various proteins with the suitable binding domains, leading to the cross-talking of series of cell signaling pathways [28, 29]. The aberrant expression of such scaffolding proteins thus are inevitably crucial to tumorigenesis and metastasis [30], for instance, Grb2 [31], 14–3-3 [32], mda-9/Syntenin [33] and p130Cas [34], which all work in such manners in various tumors. Therefore, SH3BGRL expression level could be considered as a diagnostic marker for breast tumor based on our results.

We previously showed that mouse SH3BGRL promotes cancer metastasis as a novel c-Src activator, while human SH3BGRL, as an ortholog, suppresses tumor formation and metastasis [16]. However, SH3BGRL is observed to be broadly upregulated in breast tumors, including squamous oral carcinoma, among which only rare patients contain somatic mutation, such as R76C which can mimic the mouse SH3BGRL to enhance tumorigenesis and metastasis. In contrast, low SH3BGRL expression is related to AML progression [17], indicating the dual functions of SH3BGRL in cancer progression. Considering the adaptor character of SH3BGRL, this dual-sided effect might be attributed to the specific cell contexts in different tissues, including HER2 expression state in breast tumors.

HER2-positive breast tumors account for about 22% of all breast cancers [35], which are usually metastatic with poor prognosis prior to occurrence of HER2-targeted therapy [36]. With emergence of trastuzumab (Herceptin), HER2-positive breast cancers were successfully treated [37]. However, there are patients instinctively resistant to HER2-targeted therapy, including the various HER2 mutations, hinting the incomplete understanding on HER2-positive breast tumors. Large TCGA data analyses further confirm that patients with higher SH3BGRL expression would present poor prognosis, which is in line with our results.

Functionally, Herceptin itself functions as a blocker to compete the ligand-binding site of HER2 on extracellular membrane region to inhibit HER2 activation [38], and Lapatinib binds to HER2 ATP-binding domain to block its phosphorylation and kinase activity. Our results indicated that the binding of SH3BGRL to HER2 can activate HER2 phosphorylation at Y1196 and the downstream signaling to promote tumor cell proliferation and survival, regardless of EGF stimulation, uncovering that prior to ligand stimulation, SH3BGRL may promote HER2 to form dimers with other RGFR members. Therefore, the subsequent inhibition with Herceptin or Lapatinib should be ineffective in HER2-positive breast cancers with concomitant SH3BGRL overexpression. Therefore, Targeting SH3BGRL or its downstream signaling would be a promising and effective strategy, which is also validated by our xenograft tumor therapies.

## Conclusions

Our results unravel that SH3BGRL is a novel posttranslational modulator of the inherent HER2 activation, which leads to the intrinsic resistance to HER2-targeted therapy. SH3BGRL would be a pivotal therapy target and a diagnostic marker for SH3BGRL and HER2 doubly positive cancers. In addition, interfering the downstream signaling would be an alternative and efficient therapy strategy in those breast cancers.

## Supplementary information

**Additional file 1: Table S1.** Primers for construction of the truncated mutants of HA-fused SH3BGRL. **Supplemental Figure S1.** SH3BGRL expresses in various breast cancer cell lines. **Supplemental Figure S2.** SH3BGRL prolongs HER2 stability and duration on cell membrane. **Supplemental Figure S3.** SH3BGRL promotes cell cycle progression. **Supplemental S4.** SH3BGRL renders drug resistance. **Supplemental S5.** Targeting SH3BGRL represses the xenografted tumor formation. **Supplemental Figure S6.** Targeting SH3BGRL represses cell proliferation and enhances cell apoptosis in xenografted tumors. **Supplemental Figure S7.** SH3BGRL activates HER2 to promotes tumor progression. **Supplemental Figure S8.** Relevance of SH3BGRL and HER2 in breast cancer tissues.

## Data Availability

Not applicable.

## Abbreviations

HER2: Human epidermal growth factor receptor 2
SH3BGRL: SH3 Domain Binding Glutamic Acid Rich Like
PCR: Polymerase Chain Reaction
shRNA: Short hairpin RNA
GFP: Green fluorescent protein
BSA: Bovine Serum Albumin
h: hour
CCK8: Cell Counting Kit
7-AAD: 7-amino-actinomycin D
SEM: Standard error of mean

## References

[1] Torre LA, Bray F, Siegel RL, Ferlay J, Lortet-Tieulent J, Jemal A (2015). Global cancer statistics, 2012. CA Cancer J Clin.

[2] Perou CM, Sørlie T, Eisen MB, van de Rijn M, Jeffrey SS, Rees CA, Pollack JR, Ross DT, Johnsen H, Akslen LA (2000). Molecular portraits of human breast tumours. Nature.

[3] Klapper LN, Waterman H, Sela M, Yarden Y (2000). Tumor-inhibitory antibodies to HER-2/ErbB-2 may act by recruiting c-Cbl and enhancing ubiquitination of HER-2. Cancer Res.

[4] Xu W, Marcu M, Yuan X, Mimnaugh E, Patterson C, Neckers L (2002). Chaperone-dependent E3 ubiquitin ligase CHIP mediates a degradative pathway for c-ErbB2/Neu. Proc Natl Acad Sci U S A.

[5] Dankort D, Jeyabalan N, Jones N, Dumont DJ, Muller WJ (2001). Multiple ErbB-2/Neu phosphorylation sites mediate transformation through distinct effector proteins. J Biol Chem.

[6] Dankort D, Maslikowski B, Warner N, Kanno N, Kim H, Wang Z, Moran MF, Oshima RG, Cardiff RD, Muller WJ (2001). Grb2 and Shc adapter proteins play distinct roles in Neu (ErbB-2)-induced mammary tumorigenesis: implications for human breast cancer. Mol Cell Biol.

[7] Lucs AV, Muller WJ, Muthuswamy SK (2010). Shc is required for ErbB2-induced inhibition of apoptosis but is dispensable for cell proliferation and disruption of cell polarity. Oncogene.

[8] Marone R, Hess D, Dankort D, Muller WJ, Hynes NE, Badache A (2004). Memo mediates ErbB2-driven cell motility. Nat Cell Biol.

[9] Ren Z, Schaefer TS (2002). ErbB-2 activates Stat3 alpha in a Src- and JAK2-dependent manner. J Biol Chem.

[10] Maadi H, Nami B, Tong J, Li G, Wang Z (2018). The effects of trastuzumab on HER2-mediated cell signaling in CHO cells expressing human HER2. BMC Cancer.

[11] Mazzocco M, Maffei M, Egeo A, Vergano A, Arrigo P, Di Lisi R, Ghiotto F, Scartezzini P (2002). The identification of a novel human homologue of the SH3 binding glutamic acid-rich (SH3BGR) gene establishes a new family of highly conserved small proteins related to Thioredoxin superfamily. Gene.

[12] Egeo A, Mazzocco M, Arrigo P, Vidal-Taboada JM, Oliva R, Pirola B, Giglio S, Rasore-Quartino A, Scartezzini P (1998). Identification and characterization of a new human gene encoding a small protein with high homology to the proline-rich region of the SH3BGR gene. Biochem Biophys Res Commun.

[13] Tong F, Zhang M, Guo X, Shi H, Li L, Guan W, Wang H, Yang S (2016). Expression patterns of SH3BGR family members in zebrafish development. Dev Genes Evol.

[14] Egeo A, Di Lisi R, Sandri C, Mazzocco M, Lapide M, Schiaffino S, Scartezzini P (2000). Developmental expression of the SH3BGR gene, mapping to the Down syndrome heart critical region. Mech Dev.

[15] Cesareni G, Panni S, Nardelli G, Castagnoli L (2002). Can we infer peptide recognition specificity mediated by SH3 domains?. FEBS Lett.

[16] Wang H, Liu B, Al-Aidaroos AQ, Shi H, Li L, Guo K, Li J, Tan BC, Loo JM, Tang JP (2016). Dual-faced SH3BGRL: oncogenic in mice, tumor suppressive in humans. Oncogene.

[17] Xu L, Zhang M, Li H, Guan W, Liu B, Liu F, Wang H, Li J, Yang S, Tong X, Wang H (2018). SH3BGRL as a novel prognostic biomarker is down-regulated in acute myeloid leukemia. Leuk Lymphoma.

[18] Abba MC, Hu Y, Sun H, Drake JA, Gaddis S, Baggerly K, Sahin A, Aldaz CM (2005). Gene expression signature of estrogen receptor alpha status in breast cancer. BMC Genomics.

[19] van 't Veer LJ, Dai H, van de Vijver MJ, He YD, Hart AA, Mao M, Peterse HL, van der Kooy K, Marton MJ, Witteveen AT (2002). Gene expression profiling predicts clinical outcome of breast cancer. Nature.

[20] Schulze WX, Deng L, Mann M (2005). Phosphotyrosine interactome of the ErbB-receptor kinase family. Mol Syst Biol.

[21] Yin L, Xiang Y, Zhu DY, Yan N, Huang RH, Zhang Y, Wang DC (2005). Crystal structure of human SH3BGRL protein: the first structure of the human SH3BGR family representing a novel class of thioredoxin fold proteins. Proteins.

[22] Giri DK, Ali-Seyed M, Li LY, Lee DF, Ling P, Bartholomeusz G, Wang SC, Hung MC (2005). Endosomal transport of ErbB-2: mechanism for nuclear entry of the cell surface receptor. Mol Cell Biol.

[23] Erjala K, Sundvall M, Junttila TT, Zhang N, Savisalo M, Mali P, Kulmala J, Pulkkinen J, Grenman R, Elenius K (2006). Signaling via ErbB2 and ErbB3 associates with resistance and epidermal growth factor receptor (EGFR) amplification with sensitivity to EGFR inhibitor gefitinib in head and neck squamous cell carcinoma cells. Clin Cancer Res.

[24] Li H, Sanchez-Torres J, Del Carpio A, Salas V, Villalobo A (2004). The ErbB2/Neu/HER2 receptor is a new calmodulin-binding protein. Biochem J.

[25] Nahta R, Hung MC, Esteva FJ (2004). The HER-2-targeting antibodies trastuzumab and pertuzumab synergistically inhibit the survival of breast cancer cells. Cancer Res.

[26] Shattuck DL, Miller JK, Carraway KL, Sweeney C (2008). Met receptor contributes to trastuzumab resistance of Her2-overexpressing breast cancer cells. Cancer Res.

[27] Wainberg ZA, Anghel A, Desai AJ, Ayala R, Luo T, Safran B, Fejzo MS, Hecht JR, Slamon DJ, Finn RS (2010). Lapatinib, a dual EGFR and HER2 kinase inhibitor, selectively inhibits HER2-amplified human gastric cancer cells and is synergistic with trastuzumab in vitro and in vivo. Clin Cancer Res.

[28] Deretic V (2008). Autophagosome and phagosome. Methods Mol Biol.

[29] Pawson T, Scott JD (1997). Signaling through scaffold, anchoring, and adaptor proteins. Science.

[30] Mehrotra S, Languino LR, Raskett CM, Mercurio AM, Dohi T, Altieri DC (2010). IAP regulation of metastasis. Cancer Cell.

[31] Galliher-Beckley AJ, Schiemann WP (2008). Grb2 binding to Tyr284 in TbetaR-II is essential for mammary tumor growth and metastasis stimulated by TGF-beta. Carcinogenesis.

[32] Li DQ, Wang L, Fei F, Hou YF, Luo JM, Wei C, Zeng R, Wu J, Lu JS, Di GH (2006). Identification of breast cancer metastasis-associated proteins in an isogenic tumor metastasis model using two-dimensional gel electrophoresis and liquid chromatography-ion trap-mass spectrometry. Proteomics.

[33] Boukerche H, Su ZZ, Prevot C, Sarkar D, Fisher PB (2008). mda-9/Syntenin promotes metastasis in human melanoma cells by activating c-Src. Proc Natl Acad Sci U S A.

[34] Cabodi S, Tinnirello A, Di Stefano P, Bisaro B, Ambrosino E, Castellano I, Sapino A, Arisio R, Cavallo F, Forni G (2006). p130Cas as a new regulator of mammary epithelial cell proliferation, survival, and HER2-neu oncogene-dependent breast tumorigenesis. Cancer Res.

[35] Ross JS, Slodkowska EA, Symmans WF, Pusztai L, Ravdin PM, Hortobagyi GN (2009). The HER-2 receptor and breast cancer: ten years of targeted anti-HER-2 therapy and personalized medicine. Oncologist.

[36] Gonzalez-Angulo AM, Litton JK, Broglio KR, Meric-Bernstam F, Rakkhit R, Cardoso F, Peintinger F, Hanrahan EO, Sahin A, Guray M (2009). High risk of recurrence for patients with breast cancer who have human epidermal growth factor receptor 2-positive, node-negative tumors 1 cm or smaller. J Clin Oncol.

[37] Slamon DJ, Leyland-Jones B, Shak S, Fuchs H, Paton V, Bajamonde A, Fleming T, Eiermann W, Wolter J, Pegram M (2001). Use of chemotherapy plus a monoclonal antibody against HER2 for metastatic breast cancer that overexpresses HER2. N Engl J Med.

[38] Nahta R, Yu D, Hung MC, Hortobagyi GN, Esteva FJ (2006). Mechanisms of disease: understanding resistance to HER2-targeted therapy in human breast cancer. Nat Clin Pract Oncol.

