# Supplementary material for: SH3BGRL confers innate drug resistance in breast cancer by stabilizing HER2 activation on cell membrane
Source: J Exp Clin Cancer Res. 2020 May 7;39:81. doi: 10.1186/s13046-020-01577-z (PMC7204297; doi:10.1186/s13046-020-01577-z)
Supplement: Supplementary file 1 — Additional file 1: Table S1. Primers for construction of the truncated mutants of HA-fused SH3BGRL. Supplemental Figure S1. SH3BGRL expresses in various breast cancer cell lines. Supplemental Figure S2. SH3BGRL prolongs HER2 stability and duration on cell membrane. Supplemental Figure S3. SH3BGRL promotes cell cycle progression. Supplemental S4. SH3BGRL renders drug resistance. Supplemental S5. Targeting SH3BGRL represses the xenografted tumor formation. Supplemental Figure S6. Targeting SH3BGRL represses cell proliferation and enhances cell apoptosis in xenografted tumors. Supplemental Figure S7. SH3BGRL activates HER2 to promotes tumor progression. Supplemental Figure S8. Relevance of SH3BGRL and HER2 in breast cancer tissues. [file 13046_2020_1577_MOESM1_ESM.pdf]

Table S1 Primers for construction of the truncated mutants of HA-fused SH3BGRL

Primer	Sequence
ha-SH3BGRL-F	CCGGAATTCATGGTGATCCGTGTATATATT
ha-SH3BGRL-R	CGGGATCCTCAAGCGTAATCTGGAACATCGTATGGGTATG CTTGCTGCTTTGCTTG
ha-△α1-F	ATATTGCATCTTCCTCTGGCATAGGATTTG
ha-△α1-R	CAAATCCTATGCCAGAGGAAGATGCAATAT
ha-△α2-F	AAGAAAAAGATATTGCAGCCGAAAATAGTCG
ha-△α2-R	CGACTATTTTCGGCTGCAATATCTTTTTCTT
ha-△α3-F	AGTGGATGAGAGAAAATGTACCAGCCACAG
ha-△α3-R	CTGTGGCTGGTACATTTTCTCTCATCCACT
ha-△β3-F	GTCGACCAGCCACAGGTTACAATGAAAGCC
ha-△β3-R	GGCTTTCATTGTAACCTGTGGCTGGTCGAC
ha-△α4-F	GAAAGCCAGTATCGCGGGGACGCAGTGTATG
ha-△α4-R	CATACACTGCGTCCCCGCGATACTGGCTTTC
ha-△α5-F	TTCTTTGAAGCCAGAGAAAATACAGCCCCAC
ha-△α5-R	GTGGGGCTGTATTTTCTCTGGCTTCAAAGAA
ha-△α6-F	TTGACAGCCCCACCTGGTTGA
ha-△α6-R	TCAACCAGGTGGGGCTGTCAA

Construction of the truncated HA-tagged SH3BGRL mutants was conducted with mutagenesis-based PCRs. F and R indicate the Forward and Reverse primers, respectively. The specified deleted domain of SH3BGRL are indicated with △+domain code. Primers are shown from 5’ to 3’ direction.

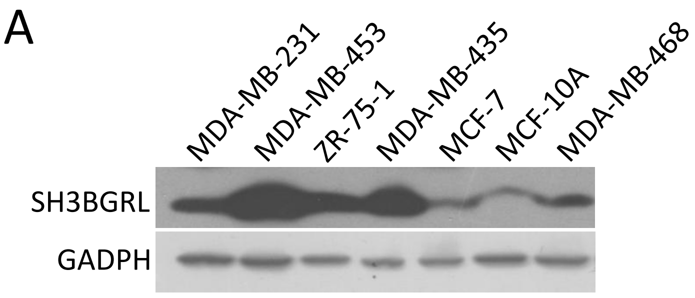

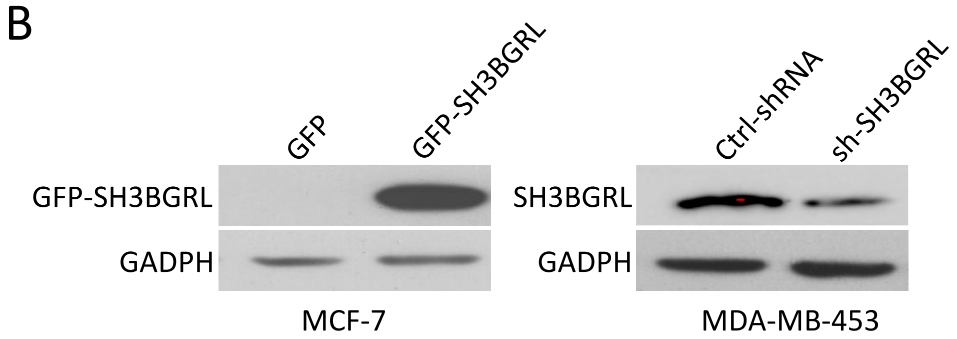

Supplemental Figure S1. SH3BGRL expresses in various breast cancer cell lines.

A. Western blots of SH3BGRL expression in human breast cancer cell lines and a normal breast cell, MCF-10A.
B. Western blots of SH3BGRL expression in MCF-7 cells with stable transfection of GFP-SH3BGRL or MDA-MB-453 cells with SH3BGRL knockdown with specific shRNAs' mixture.

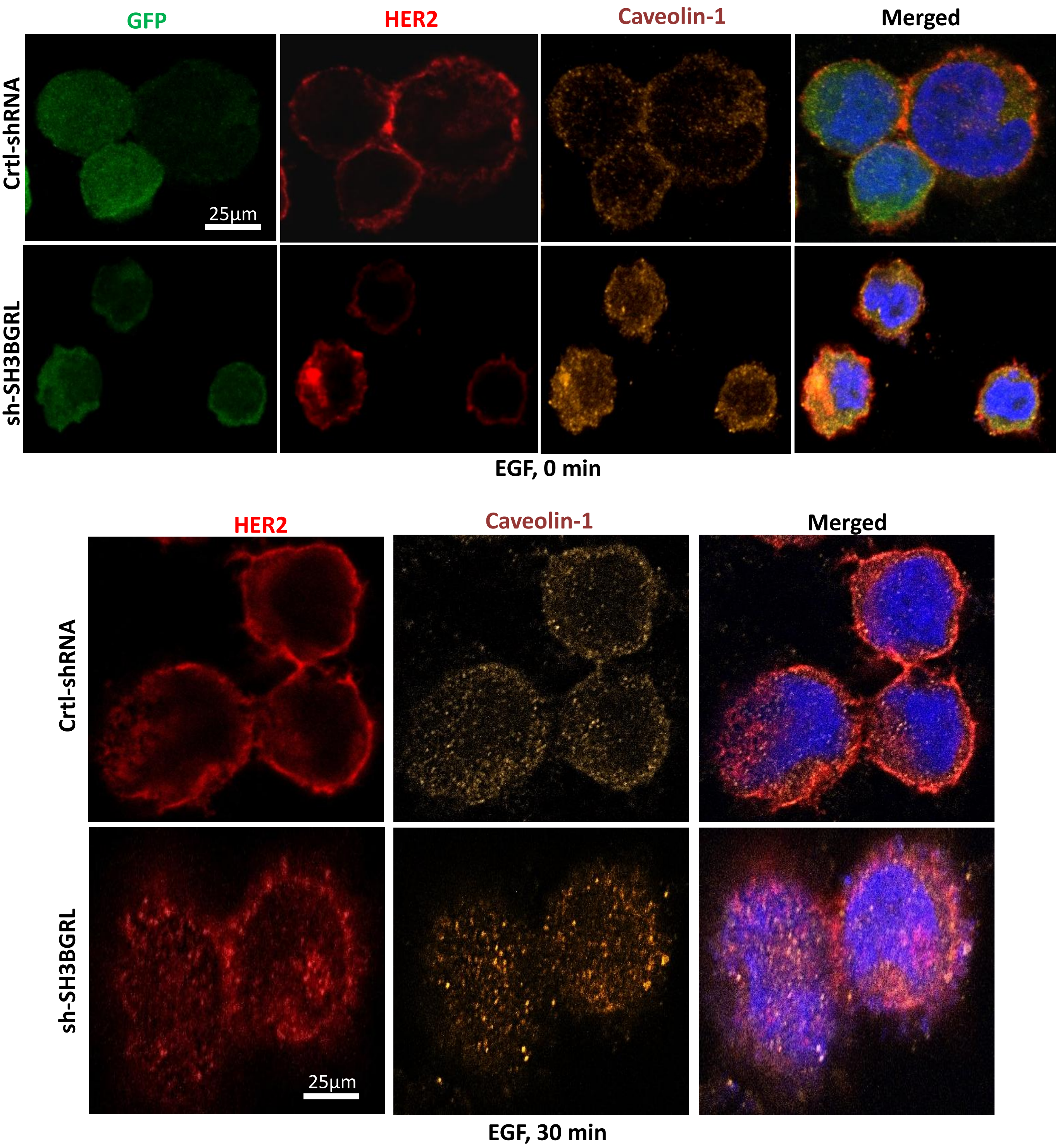

Supplemental Figure S2. SH3BGRL prolongs HER2 stability and duration on cell membrane. Immunofluorescence of the co-localization of HER2 with endocytosis-related Caveolin-1 in MDA-MB-453 cells. Cells were stained with Caveolin-1 (yellow), HER2 antibody (red), and the merged images are shown. With the treatment of EGF for 30 min, HER2 are still maintained on cell membrane, whereas with SH3BGRL knockdown , the apperant endocytosis is observed.

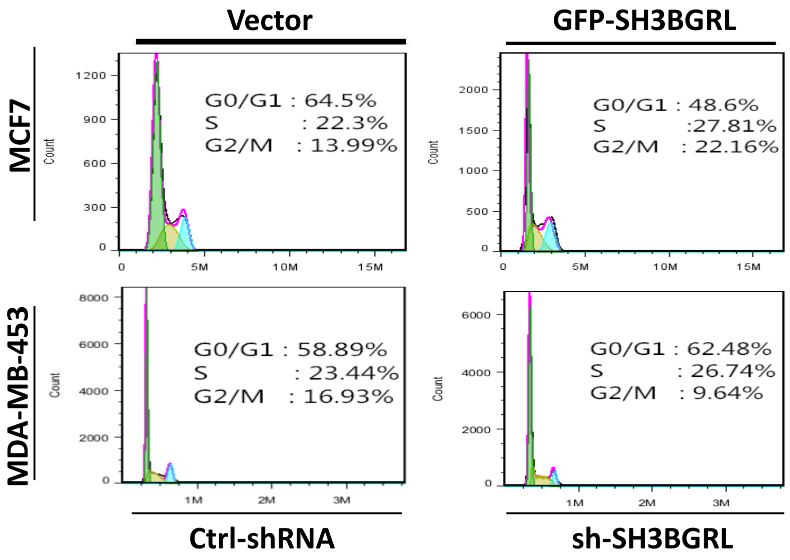

Supplemental Figure S3. SH3BGRL promotes cell cycle progression.

Representative flow cytometry histograms of cell cycle distribution in MCF-7 and MDA-MB-453 cells with SH3BGRL overexpression or knockdown cells, compared to their correspondingly parental cells, respectively,.

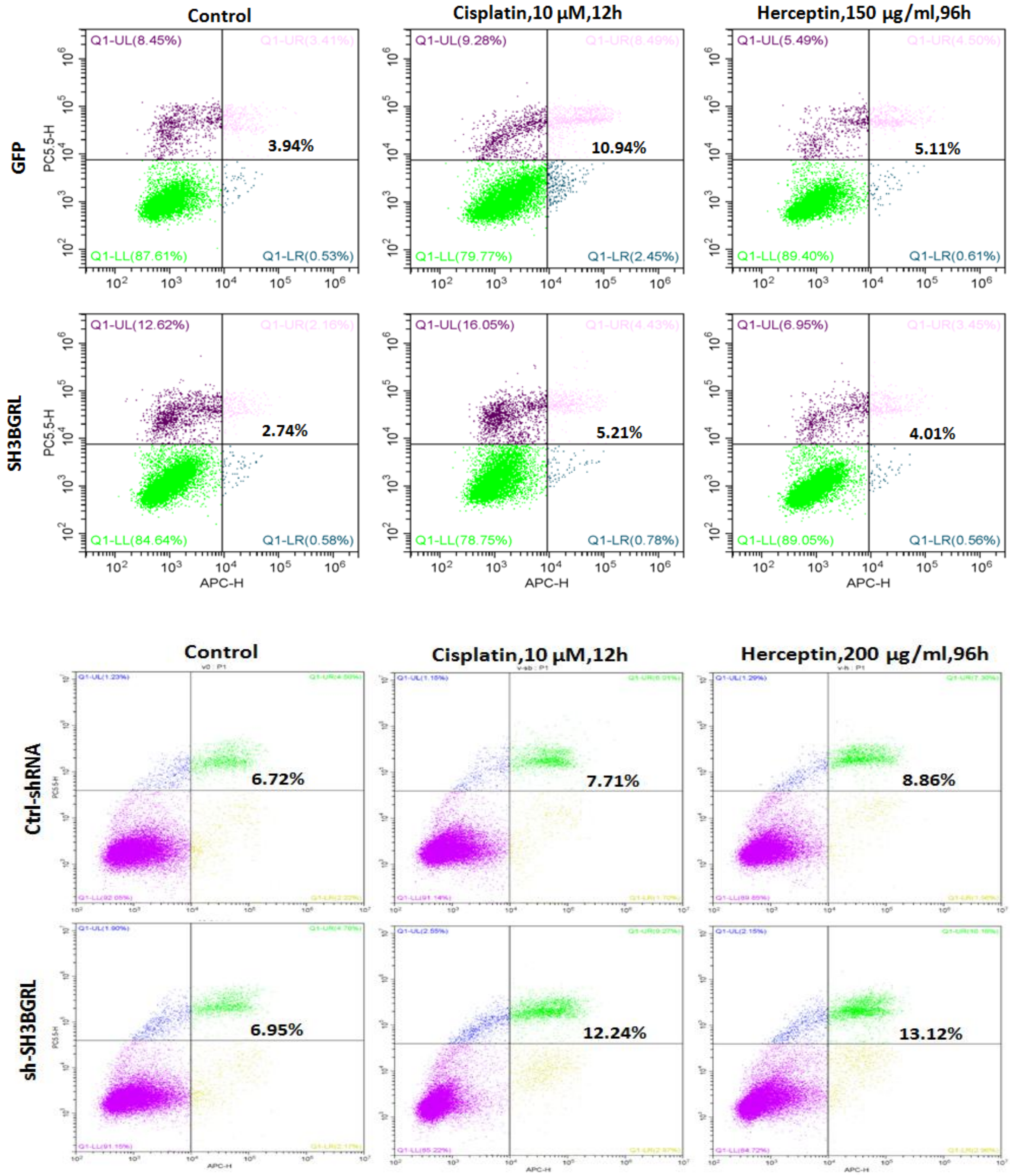

Supplemental S4. SH3BGRL renders drug resistance.

Representatives of the flow cytometry of apoptotic cells in the SH3BGRL-overexpressing or knockdown MCF-7 and MDA-MB-453 cells, respectively, compared to their counterparts upon the indicated dosages of Cisplatin or Herceptin treatment.

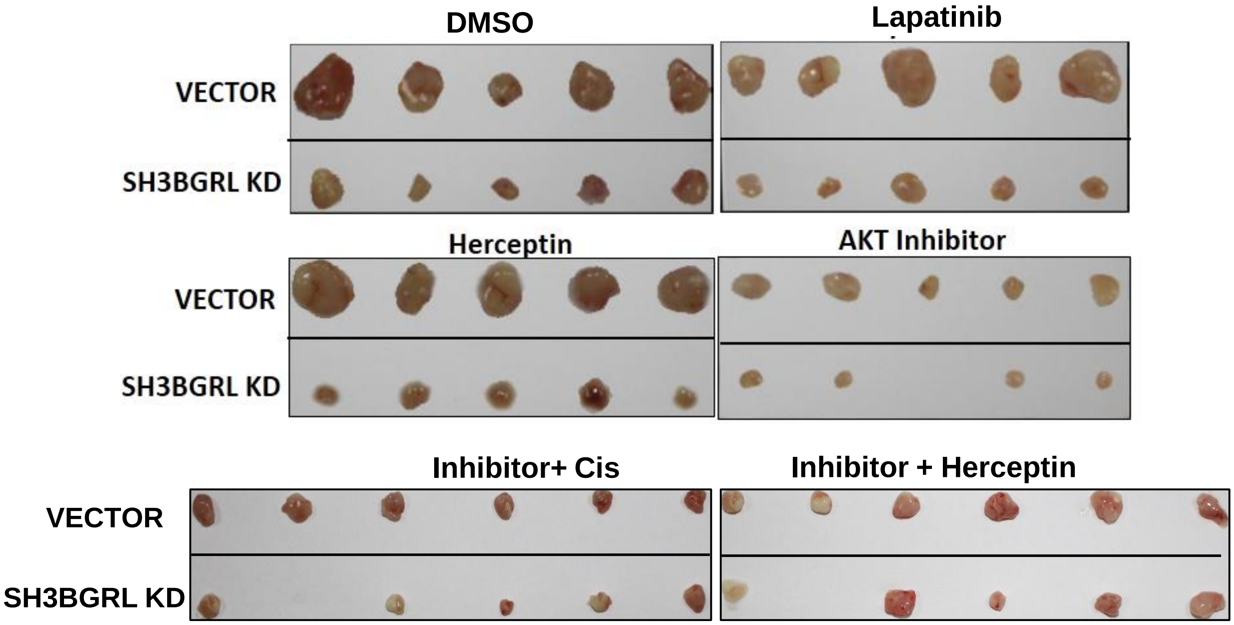

Supplemental S5. Targeting SH3BGRL represses the xenografted tumor formation

Photographs of the formed tumors induced by MDA-MB-453 cells (Vector) and SH3BGRL knockdown cells (SH3BGRL KD) upon Lapatinib, Herceptin and LY294002 (Inhibitor) or their combination treatments. DMSO was used as a blank control.

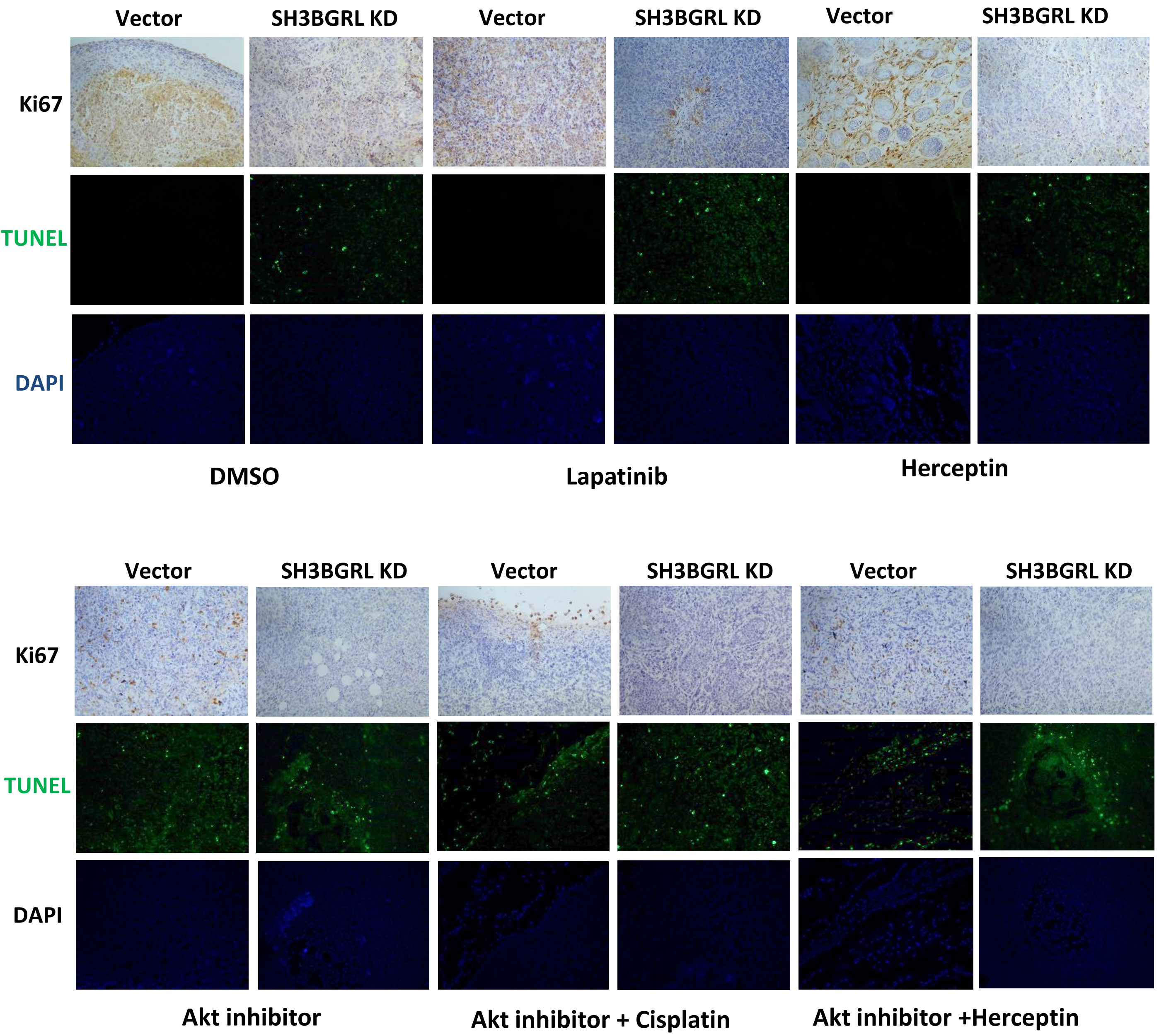

Supplemental Figure S6. Targeting SH3BGRL represses cell proliferation and enhances cell apoptosis in xenografted tumors.

Ki67 staining and TUNEL apoptosis analyses were used to detect the cell proliferation and apoptosis states of the xenografted tumors. DAPI was used to counterstain the cell nucleus. All representative pictures were took under 200X magnification.

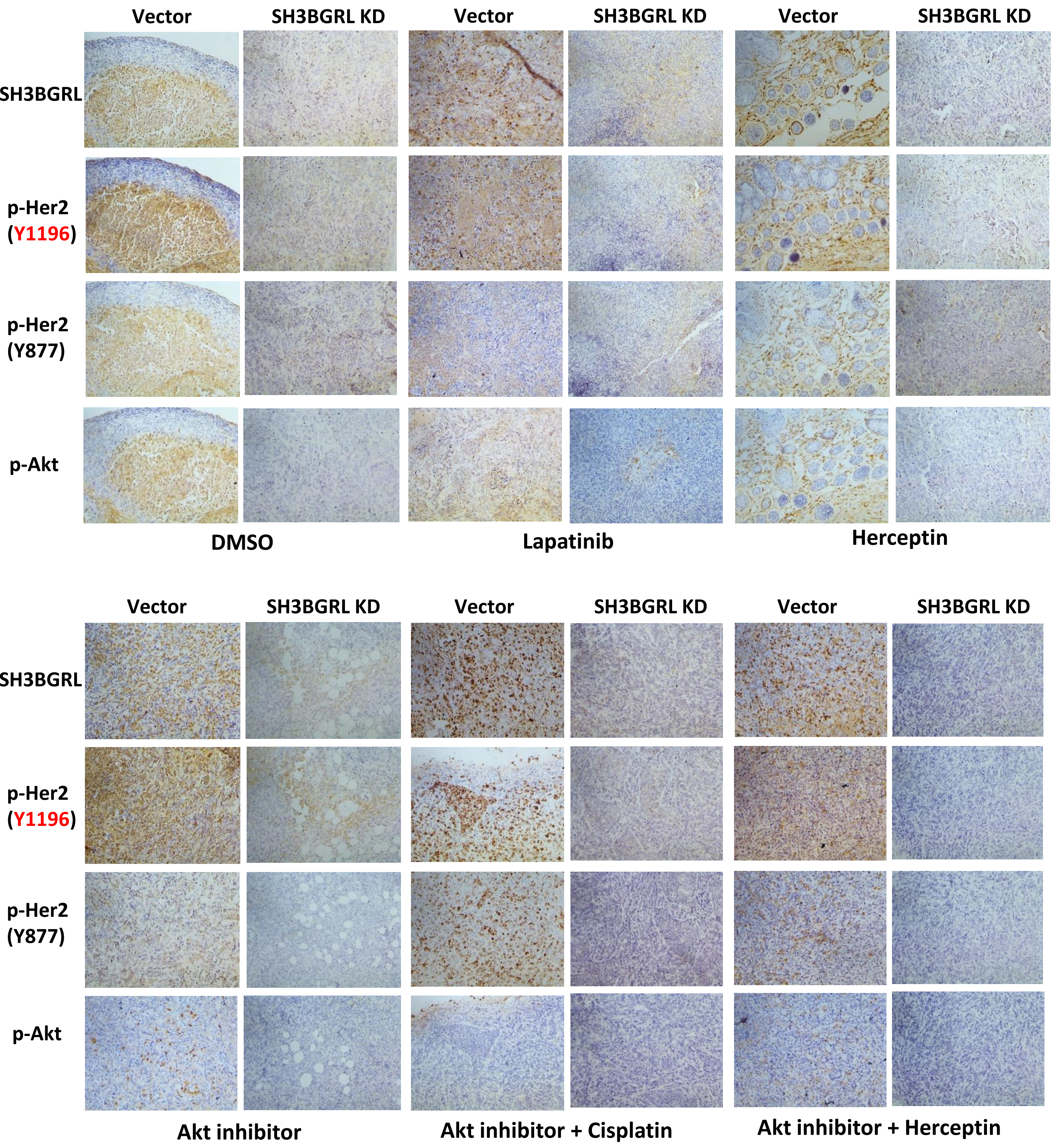

Supplemental Figure S7. SH3BGRL activates HER2 to promotes tumor progression.

Immunohistochemistry staining of the in vivo formed tumors from the indicated parental MDA-MB-453 cells (Vector) or SH3BGRL knockdown cells (SH3BGRL KD). Expressions of SH3BGRL, p-Her2(Y1196), p-Her2(Y877) and phospho-Akt were detected. Tumor groups were divided with Lapatinib, Herceptin, LY294002, LY294002+Cisplatin, and LY294002+Herceptin treatments. DMSO was used as a drug-free control. All representative pictures were took under 200X magnification.

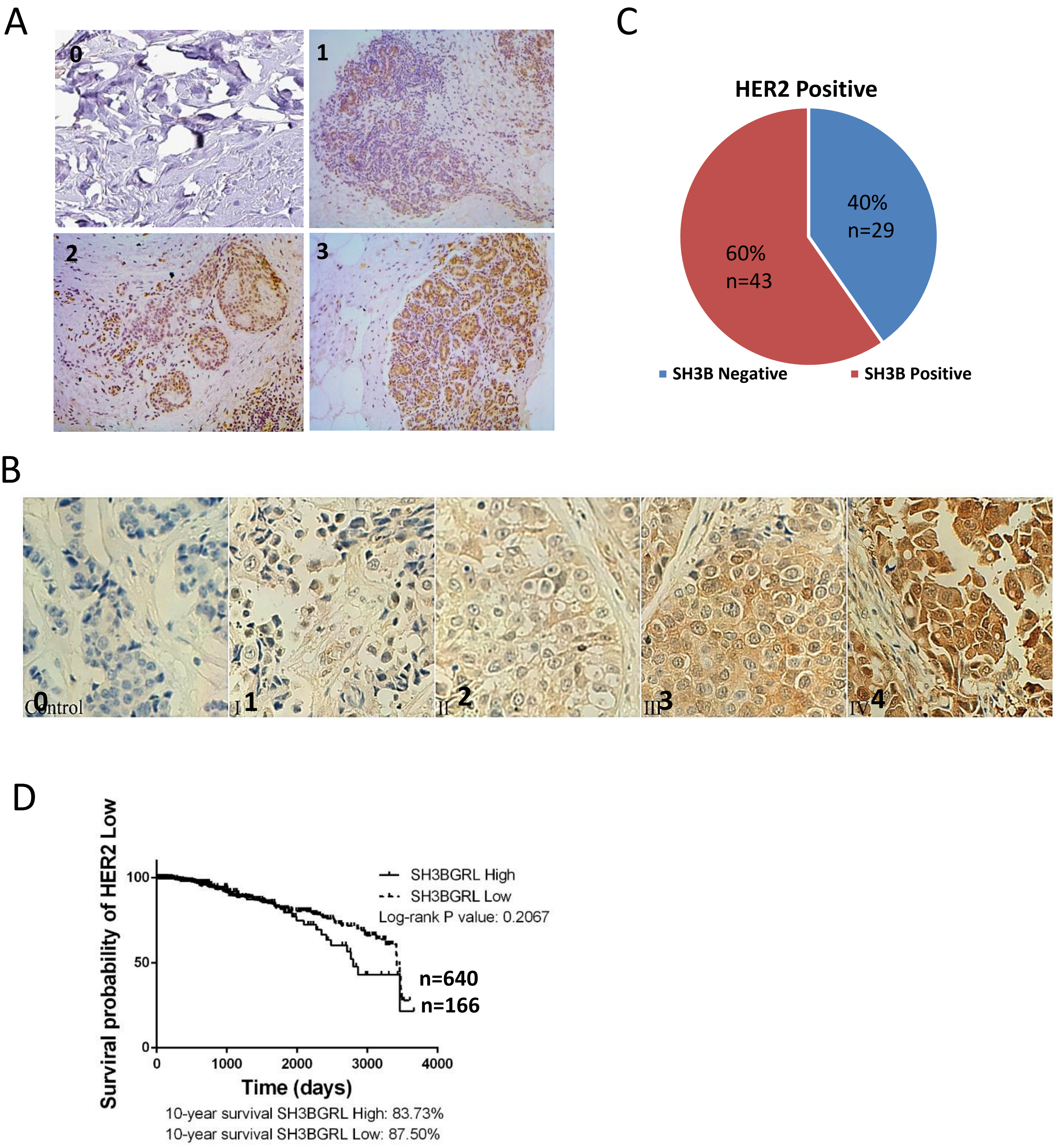

Supplemental Figure S8. Relevance of SH3BGRL and HER2 in breast cancer tissues

A. Representative IHC staining intensities of SH3BGRL or pHER2 (Y1196) expression levels. Four grades, 0,1,2 and 3 were scored for the fresh patient tissues.

B. Representative IHC staining intensities of SH3BGRL expression levels in breast cancer tissue microarrays. Five grades, 0,1,2,3, and 4 were scored.

C. The percentage of SH3BGRL-negative or -positive cases in HER2-positive tissues.

D. Kaplan-Meier survival analyses of 806 patients with low HER2 expression from TCGA database. There is no survival difference between the SH3BGRL-high and -low groups in HER2-low cases.
